# Primary renal lymphoma: an unusual finding following radical nephrectomy 

**DOI:** 10.5414/CNCS108955

**Published:** 2017-01-31

**Authors:** Cody M. Rissman, Lawrence M. Dagrosa, Jason R. Pettus, Jessica L. Dillon, Einar F. Sverrisson

**Affiliations:** 1 Dartmouth – Geisel School of Medicine, Hanover, and; 2 Dartmouth-Hitchcock Medical Center, Lebanon, NH, USA

**Keywords:** lymphoma, kidney

## Abstract

Secondary kidney involvement by disseminated non-Hodgkin’s lymphoma (NHL) is quite common and is estimated to approach 30 – 60% in NHL patients. However, primary renal lymphoma is exceedingly rare and estimated to make up less than 1% of all kidney masses. We report a case of primary renal NHL presenting with profound hypercalcemia and renal failure recalcitrant to medical management, ultimately treated with urgent radical nephrectomy. To our knowledge, this is the first report of primary renal lymphoma presenting in this acute fashion.

## Case history 

A 54-year-old woman presented to the emergency department with self-reported new onset headache, sensitivity to light, intermittent blurred vision, mental dullness, and slurred speech of 3-days duration. 

On examination, the patient was afebrile and hypertensive with a blood pressure of 166/86, which was noted to be significantly elevated over her baseline. She was alert and oriented, and her neurological exam was non-focal. Imaging of the brain showed no abnormalities. Initial labs were concerning for a serum calcium of 14.3 mg/dL, creatinine of 4.54 mg/dL (eGFR 10), blood urea nitrogen of 50 mg/dL, serum phosphorus of 5.9 mg/dL. Other electrolytes and liver function tests were all unremarkable. Her white blood cell count was 5,100/µL, hemoglobin 10.7 g/dL, hematocrit 31.5% with an unremarkable differential. Urinalysis demonstrated a trace amount of blood with no protein, glucose, ketones, or nitrites. Serum and urine protein electrophoresis were unremarkable. A large non-tender mass was palpable in the right upper quadrant. 

**Figure 1.**
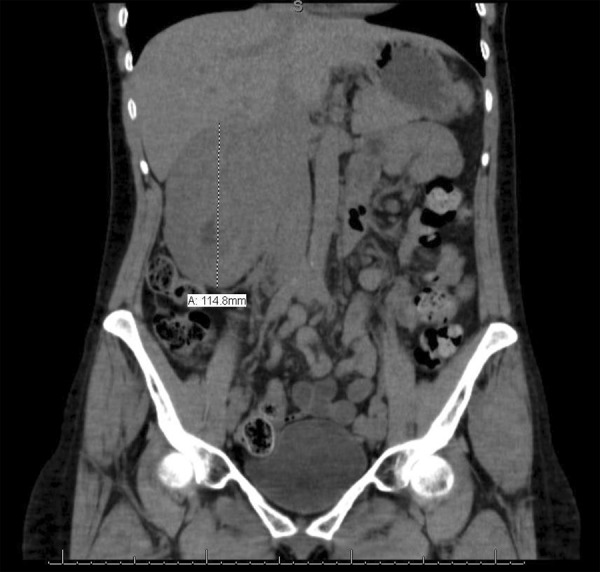
CT abdomen – pelvis.

Computed tomography (CT) of the abdomen and pelvis was obtained. This demonstrated a large heterogeneous right renal mass with suggestion of active bleeding consistent with a hemorrhagic renal cell cancer (Figure 1). Chest radiography was normal. 

Further history revealed no recent weight loss, fever, dysuria, flank pain, hematuria, urinary retention, or lower urinary tract symptoms. The patient reported a remote history of urethral stricture dilation as a child and several episodes of urinary retention and failed voiding trials following urethral catheterization. She also reported urinary frequency with hourly voiding since childhood. There was no known family history of urological cancer. 

A standard workup for hypercalcemia including 25-OH vitamin D and 1,25 vitamin D levels, parathyroid hormone, parathyroid hormone related protein, and thyroid function tests was ordered. All values were within normal limits with the exception of an elevated 1,25 vitamin D of 90 pg/mL (reference range 18 – 78 pg/mL). 

The patient’s hypercalcemia was treated with volume expansion and furosemide, but after 6 days her metabolic derangements remained poorly controlled. As expected, a mercaptoacetyltriglycine renal scan demonstrated global bilateral renal dysfunction with the patient’s normal kidney contributing 96% of her remaining renal function. Non-contrast abdominal magnetic resonance imaging confirmed a right renal mass measuring ~ 11 × 8 × 11.5 cm with areas of hemorrhage throughout and no suggestion of renal vein thrombus. A metastatic evaluation was negative. It was felt that a paraneoplastic phenomenon was driving the patient’s renal failure and metabolic derangements. A multidisciplinary review of her case by nephrology, urology, and hospital medicine services resulted in a decision to pursue a therapeutic and diagnostic urgent radical nephrectomy. An uncomplicated open right radical nephrectomy was performed on hospital day 8 with a suspicious intra-aortocaval lymph node removed. The patient tolerated the procedure well and her electrolytes and renal function normalized within 48 hours of surgery. 

**Figure 2.**
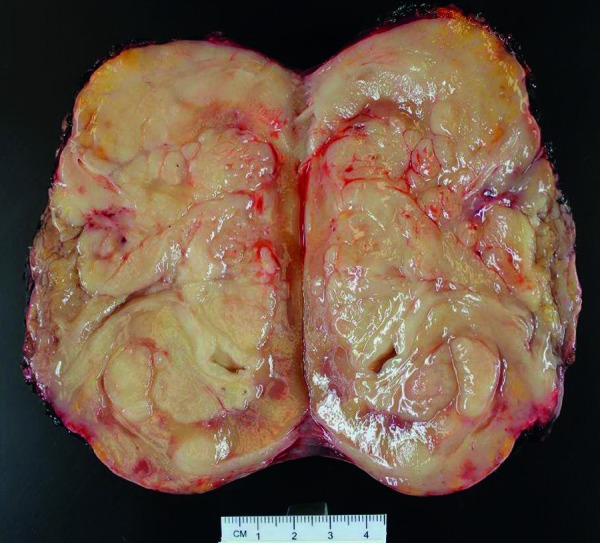
Gross pathology demonstrates near diffuse replacement of bisected renal parenchyma by infiltrative tumor, extending into perinephric adipose tissue. The proximal ureteral wall (left) is markedly thickened and involved by lymphoma.

**Figure 3.**
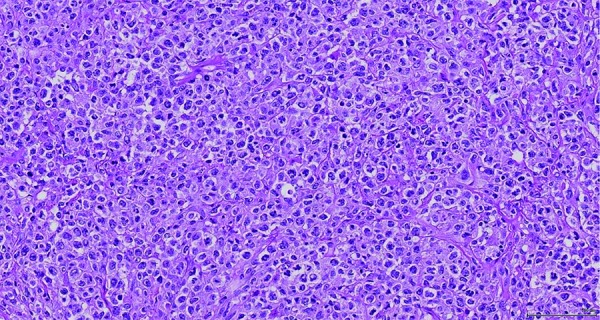
H & E slide 200×. Diffusely infiltrative diffuse large B-cell lymphoma.

**Figure 4.**
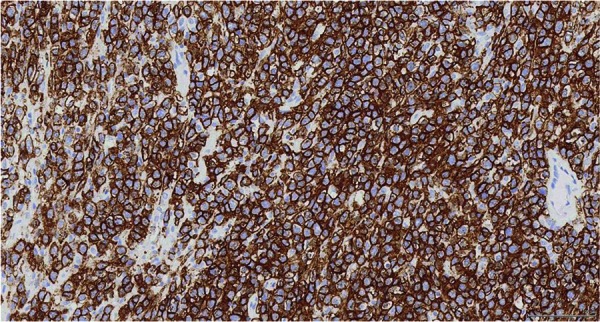
CD20 IHC 200×. Diffuse membranous staining for CD20, consistent with B-cell origin.

The pathologic specimen grossly showed a firm, diffusely infiltrative mass occupying the majority of the renal parenchyma with gross extension into perinephric adipose tissue (Figure 2). Microscopy of the mass revealed a population of large cells with atypical nucleoli, abundant mitoses, and prominent clear to eosinophilic cytoplasm (Figure 3). Some regions of tumor cells appeared epithelioid, and the consideration of a poorly differentiated renal cell carcinoma was originally considered. An immunohistochemistry panel, however, revealed the following phenotype: CD20+, CD10+, BCL6+. This confirmed the diagnosis of diffuse large B-cell lymphoma (DLBCL) (Figure 4). The lymphoma was diffusely infiltrative into the wall of the renal artery and renal vein, renal pelvis, ureteral margin, perinephric fat, and adrenal gland capsule. An aorto-caval lymph node removed at the time of nephrectomy demonstrated reactive lymph node hyperplasia only, with no evidence of lymphoma. 

The patient subsequently underwent a bone marrow staging biopsy which showed normal trilineage maturation, negative for lymphoma. The patient’s initial chemotherapy regimen consisted of rituximab, cyclophosphamide, doxorubicin hydrochloride, vincristine sulfate, and prednisone (R-CHOP). 

## Discussion 

Unilateral renal masses can be malignant, benign, or inflammatory in nature. Malignant renal tumors include renal cell carcinoma, urothelium-based malignancies, sarcomas, embryonal tumors, lymphoma, and metastases. Commonly identified benign renal masses include cystic lesions, angiomyolipomas, and oncocytomas among others. Inflammatory renal masses such as abscesses, tuberculosis, and focal pyelonephritis can also present with unique diagnostic challenges. CT with and without contrast has become the most important radiographic test for evaluation of renal masses. Contrast is used to take advantage of enhancement characteristics of highly vascular renal parenchymal tumors. CT findings are inconclusive, however, in ~ 10 – 20% of cases, thus necessitating additional imaging, biopsy, or surgery to confirm a diagnosis. Renal mass biopsy may be of use in patients who are potential candidates for a wide array of treatment options. Traditionally, percutaneous renal biopsy of a renal mass is indicated when there is high suspicion that the mass is a metastatic lesion, lymphoma, or an abscess [1]. 

The presence of renal involvement secondary to disseminated disease is estimated to ~ 30 – 60% in non-Hodgkin’s lymphoma (NHL) patients [2, 3, 4, 5]. However, primary renal lymphoma (PRL), defined as NHL arising in the renal parenchyma and not from invasion of an adjacent lymphomatous mass, is estimated to represent less than 1% of all kidney masses [3]. The existence of PRL has long been disputed as the kidneys are devoid of lymphatic tissues. Although the actual number of reported PRL cases is questionable secondary to incomplete staging and the presence of extrarenal involvement at the time of diagnosis, an accumulation of new literature in recent years suggests that the entity of PRL does exist [4, 5, 6]. There are several theories surrounding the origin of PRL. One proposed mechanism suggests that chronic inflammation of the kidney leads to the invasion of lymphoid cells that then undergo oncogenic transformation in situ [4, 5]. Similar pathogenesis has been well documented in cases of lymphoma arising in both Hashimoto’s thyroiditis and *H. pylori* gastritis in the thyroid and stomach, respectively [4, 7, 8]. Cupisti et al.’s [4] report of PRL arising in a patient with chronic pyelonephritis supports the inflammatory nidus theory; however, this phenomenon is not well documented in other case reports including our own. Others speculate that these lymphomas arise in the lymphatic channels surrounding the renal capsule and progress to infiltrate the renal parenchyma. 

Preoperative diagnosis of PRL is rare and is likely secondary to its lack of specific clinical symptoms at presentation. Kandel et al. [5] reviewed 28 cases of PRL and found that 17 patients had extrarenal disease at time of autopsy. The question of whether cases such as these are evidence of rapid metastasis of primary tumor of the kidney or if renal involvement is secondary to disseminated disease remains difficult to answer. Thus, Stallone et al. [4] and others have proposed the following diagnostic criteria for PRL: 1) lymphomatous renal infiltration, 2) non-obstructive unilateral or bilateral kidney enlargement, 3) no extrarenal localization at the time of diagnosis verified by thoracoabdominal CT, bone marrow biopsy, and renal biopsy. The most common presenting signs and symptoms reported in the current literature include pain, abdominal mass, anorexia, vomiting, fever, hypertension, and acute renal injury [2, 3, 4, 5, 6, 9]. The presence of hypercalcemia in PRL at presentation does not appear to be common. Our case, as well as the case reported by Cupisti et al. [6], was associated with an elevated serum 1,25 OH vitamin D. The most common cause of hypercalcemia in a hospitalized patient is cancer. Hypercalcemia of malignancy can be caused by osteolytic metastases or by the production of parathyroid-related hormone or 1,25-dihydroxyvitamin D (calcitriol). Overproduction of calcitriol is responsible for the vast majority of cases of hypercalcemia in Hodgkin’s lymphoma and ~ 1/3 of cases in NHL [10, 11]. 

Acute kidney injury (AKI) secondary to PRL is likely secondary to infiltration of the renal interstitium by monomorphic lymphoid cells. High tumor burden has been shown to cause impairment of vascularization through obliteration of the intertubular capillary network and disruption of the normal architecture of the renal tubules and glomeruli. Isolated interstitial involvement may help to explain the absence of protein or tumor cells in the urine and a frequently normal urinalysis [3, 4]. Additionally, the presence of profound hypercalcemia, as in our case, can exacerbate or cause AKI through renal vasoconstriction and intratubular calcium deposition. This was likely the primary cause of AKI in our report, as renal function normalized with nephrectomy and correction of the hypercalcemia. 

Histologies at presentation are often intermediate to high grade, and prognosis is poor with a median survival of less than 1 year [3, 5]. Due to the low incidence of PRL, randomized controlled trials have not been developed to assess treatment strategies. Currently, systemic chemotherapy with or without radiotherapy is the most widely accepted treatment. The high prevalence of death within the first year has been attributed to rapid relapse and infection during the course of neutropenia. Those with unilateral disease treated with surgical resection and combined chemotherapy have been shown to have longer disease-free periods and better overall survival [6]. Cure has been documented in patients who underwent resection, combination chemotherapy, and consolidation radiotherapy [3]. 

## Conflicts of interests 

The authors of this manuscript declare no conflicts of interest. 

## References

[1] Campbell SC Brian LR Malignant Renal tumors In: Campbell-Walsh Urology. 11th edition. Philadelphia, PA: Elsevier; 2016 p. 1314-1364.

[2] Wang Y Guo S Primary renal diffuse large B-cell lymphoma with central nervous system involvement: a rare case report and literature review. Int J Clin Exp Pathol. 2015; 8: 7045–7049. 26261597 PMC4525931

[3] Okuno SH Hoyer JD Ristow K Witzig TE Primary renal non-Hodgkin’s lymphoma. An unusual extranodal site. Cancer. 1995; 75: 2258–2261. 7712433 10.1002/1097-0142(19950501)75:9<2258::aid-cncr2820750911>3.0.co;2-s

[4] Stallone G Infante B Manno C Campobasso N Pannarale G Schena FP Primary renal lymphoma does exist: case report and review of the literature. J Nephrol. 2000; 13: 367–372. 11063141

[5] Kandel LB McCullough DL Harrison LH Woodruff RD Ahl ET Munitz HA Primary renal lymphoma. Does it exist? Cancer. 1987; 60: 386–391. 3594375 10.1002/1097-0142(19870801)60:3<386::aid-cncr2820600317>3.0.co;2-4

[6] Cupisti A Riccioni R Carulli G Paoletti S Tognetti A Meola M Francesca F Barsotti G Petrini M Bilateral primary renal lymphoma treated by surgery and chemotherapy. Nephrol Dial Transplant. 2004; 19: 1629–1633. 15150359 10.1093/ndt/gfh250

[7] Aozasa K Inoue A Tajima K Miyauchi A Matsuzuka F Kuma K Malignant lymphomas of the thyroid gland. Analysis of 79 patients with emphasis on histologic prognostic factors. Cancer. 1986; 58: 100–104. 3708539 10.1002/1097-0142(19860701)58:1<100::aid-cncr2820580118>3.0.co;2-1

[8] Parsonnet J Hansen S Rodriguez L Gelb AB Warnke RA Jellum E Orentreich N Vogelman JH Friedman GD Helicobacter pylori infection and gastric lymphoma. N Engl J Med. 1994; 330: 1267–1271. 8145781 10.1056/NEJM199405053301803

[9] Das RN Dasgupta S Mondal S Saha D Chatterjee U Diffuse large B-cell lymphoma of the kidney: a rare neoplasm. Indian J Pathol Microbiol. 2013; 56: 449–452. 24441246 10.4103/0377-4929.125368

[10] Luceri PM Haenel LC A challenging case of hypercalcemia. J Am Osteopath Assoc. 2013; 113: 490–493. 23739761

[11] Seymour JF Gagel RF Calcitriol: the major humoral mediator of hypercalcemia in Hodgkin’s disease and non-Hodgkin’s lymphomas. Blood. 1993; 82: 1383–1394. 8364192

